# Evaluation of effects of curcumin on acute esophagitis in the corrosive esophagitis model in rats

**DOI:** 10.1007/s00210-024-03038-2

**Published:** 2024-03-18

**Authors:** Ismail K. Gurlek, Ahmet Muderrisoglu, Zafer C. Er, Akgul Arici, Mustafa Kupeli

**Affiliations:** 1 Outpatient Clinic for Thoracic Surgery, Ministry of Health, Bilecik State Hospital, Bilecik, Turkey; 2 Department of Pharmacology, Faculty of Medicine, Kırıkkale University, Kırıkkale, Turkey; 3 Department of Cardiovascular Surgery, Faculty of Medicine, Yozgat Bozok University, Yozgat, Turkey; 4 Department of Pathology, Faculty of Medicine, Gaziosmanpasa University, Tokat, Turkey; 5 Department of Thoracic Surgery, Faculty of Medicine, Yozgat Bozok University, Yozgat, Turkey

**Keywords:** Burns, Corrosion, Curcumin, Esophagitis, Inflammation

## Abstract

Ingestion of a corrosive substance may cause corrosive esophagitis. Curcumin has anti-inflammatory and mucosal protective effects. In this study, the effects of curcumin on the acute phase of corrosive esophagitis were investigated. Twenty-seven Wistar Albino rats were divided into four groups; sham (group I), control (group II), and experiment groups (group III, 100 mg/kg curcumin; group IV, 200 mg/kg curcumin). Forty percent sodium hydroxide solution was used to erode the esophagi of rats in groups other than the sham group. Curcumin was applied to animals in the experiment groups 10 min after the corrosion. After 24 h, animals were sacrificed, and esophagus samples were collected. According to the histopathological examination, the muscularis mucosa damage was regressed from 100% in group II to 71.4% in group III and 50% in group IV. Mild level of damage and collagen deposition in the tunica muscularis regressed from 66.7% of the animals in the control group to 42.9% in group III and to none in group IV. Further, an increase in submucosal collagen was present in all samples from groups II and III, while 83.3% of samples had an increase in submucosal collagen in group IV. There was a significant difference in the histopathological total score between the control group and group IV (*p*=0.02). The results showed that the administration of curcumin in a dose-dependent manner can relieve the acute phase of corrosive esophagitis.

## Introduction

Corrosive esophageal burn causes rapid tissue damage and treatment of its complications can sometimes be difficult. These burns mostly occur after accidental ingestion of corrosive substances in infants and intentional ingestion of the same in adults to commit suicide. The level of tissue damage depends on the pH, type, density, quantity, and contact time of the ingested corrosive substance and the location of the burned area (Millar and Cox 2015). Unlike acids, alkaline compounds are tasteless and odorless which make them easy to swallow without triggering protective reflexes such as vomiting (Peters and De Meester 1999). Therefore, corrosive substances that cause burns in the esophagus are usually alkaline in nature. A 2017 study that investigated toxicity of oven cleaners found that most exposure was resulted from an ingestion of sodium hydroxide or potassium hydroxide containing solution and 41.1% of the patients developed symptoms (Day et al. 2017). Alkaline compounds are capable of damaging lipoprotein membranes in all layers of the esophagus in a short time by causing liquefactive necrosis which is characterized by inflammation and liquefaction of the tissues (Peters and De Meester 1999).

On the other hand, supportive care remains the choice for treating esophageal burns with no perforation, and there has been no conclusive treatment protocol established yet (O’Malley and O’Malley 2023).

Curcumin is obtained from turmeric, which is used as a spice. It has anti-inflammatory, anti-mutagenic, anti-metastatic, and antioxidant effects and it regulates immune response (Fabianowska-Majewska et al. 2021). Curcumin exerts its beneficial effects through downregulating NLR family pyrin domain containing 3 (NLRP3) inflammasomes; suppressing nuclear factor kappa-B signaling pathway; decreasing cyclooxygenase‐2 (COX-2) activity; and reducing the levels of proinflammatory cytokines such as interleukin (IL)‐1β, IL‐6, IL‐18, and tumor necrosis factor-alpha (TNF‐α) (Karimian et al. 2017). There are animal studies in the literature indicating that curcumin can be of use against some conditions characterized by inflammation and ischemia. A previous study that applied curcumin daily to Wistar rats for 1 week found that curcumin dose-dependently reduced the levels of IL‐1β, TNF-α, and elastase and ameliorated the symptoms especially at a dose of 200 mg/kg in a monosodium urate (MSU) induced animal model of gout (Li et al. 2019). Moreover, Li et al. demonstrated that intraperitoneal (i.p.) injection of curcumin produced a neuroprotective effect against ischemic brain injury and neurological deficits in a rat model of ischemic stroke (Li et al. 2015). On the other hand, Gong et al. showed that daily i.p. administration of curcumin diminished the levels of proinflammatory cytokines such as IL-1β, IL-6, and monocyte chemoattractant protein-1 and reduced the activity of MPO in dextran sulphate sodium (DSS) induced colitis mice model. They also found that curcumin decreased the severity of histological colitis in colon tissues. The research suggested that curcumin might potently inhibit the activity of DSS-induced NLRP3 inflammasomes by modulating ROS formation, potassium efflux, and release of cathepsin B, thus showing a mucosal protective effect against colitis (Gong et al. 2018). As can be understood from the results of the mentioned studies, curcumin alleviates the effects of inflammation and ischemia in tissues, including the gastrointestinal mucosa. However, curcumin’s effect on esophageal tissue damage is yet to be investigated.

We hypothesized that curcumin decreases the level of tissue damage in early phase corrosive esophagitis caused by an alkaline substance, which is characterized by inflammation and necrosis. In this study, we aimed to investigate the effect of curcumin against esophageal burns caused by an alkaline corrosive substance in the acute phase.

## Materials and methods

### Experiment groups

All institutional and national guidelines for the care and use of laboratory animals were followed. Ethical approval for the study was obtained from Gaziosmanpasa University Animal Experimentations Ethics Board (Protocol No: HADYEK-37). Twenty-seven 6-months-old Wistar Albino rats were used. Experiment groups are shown in Table 1. The rats were kept in a temperature-controlled environment under light/dark cycles and provided ad libitum access to food and water. Rats were starved for 12 h. Anesthesia was done by using 50 mg/kg ketamine (Ketalar, Pfizer) and 10 mg/kg xylazine (Alfazyne, Alfasan) mixture via a subcutaneous route. Following the anesthetization, the route between the stomach and esophagus was obstructed by using a 2 mm width Foley catheter, while rats were in a 60% horizontal position to prevent aspiration. Forty percent sodium hydroxide (NaOH) (Merck KGaA, Darmstadt, Germany) was used to erode esophagi as described by a previous method (Senturk et al. 2010). A 0.3 ml of the solutions were applied for 60 s. For group I, applied solution was saline, while 40% NaOH solution was given in groups II, III, and IV. Because it has been shown that 10 min of caustic substance exposure is sufficient to provoke esophagus necrosis (Mattos et al. 2006), we preferred to wait for 10 min after corrosive substance application. Afterwards, saline was administered via i.p. route to rats in the groups I (sham group) and II (control group), while i.p. 100 mg/kg dose of curcumin was applied to rats in group III, and i.p. 200 mg/kg dose of curcumin was applied to rats in group IV. Curcumin powder (Sigma-Aldrich, St. Louis, Missouri, USA) was stored at -20 °C until use and dissolved in a 95% saline-5% ethanol solution before administration. Curcumin doses and administration route were selected from previous similar studies in the literature (Li et al. 2019). Morphine (Morphine HCI, Galen) was used for analgesia. One rat was excluded from groups II and IV because of premature death resulting from distal esophagus perforation and hemorrhage. After 24 h, animals were euthanized by using carbon dioxide. Rats were given i.p. 10 ml of saline to prevent hypovolemia during the 24 h between the experiment and the euthanasia.

**Table 1:** Experiment groups

Group	n	Explanation
I (Sham)	6	Rats which saline was applied to their esophagus lumen instead of a corrosive substance and received saline intraperitoneally 10 min after
II (Control)	7	To corrode the esophagus, 40% NaOH solution was applied to the esophagus lumen in this group. Saline was given intraperitoneally 10 min after esophagus corrosion. One rat was died prematurely because of a distal esophagus perforation and was excluded from the study
III	7	Rats which received intraperitoneal 100 mg/kg dose of curcumin 10 min after the corrosion of the esophagus with 40% NaOH solution
IV	7	Rats which received 200 mg/kg curcumin intraperitoneally 10 min after esophagus corrosion. One rat died prematurely and as a result excluded from the study

### Histopathological examination

Samples were collected from the burned esophagus area via serial sectioning. Samples were prepared as paraffin blocks. Blocks were prepared in 5-µm thickness of transverse blocks and then stained with hematoxylin-eosin (H&E). They were also stained with Masson’s trichrome (MT) to evaluate connective tissue changes. Levels of tissue damage to samples were evaluated by using a previously established histopathological method with a 5-point scoring system that is described in Table 2 (Turkyilmaz et al. 2005). Histopathological evaluation was performed by a pathologist who was blinded to the samples.

**Table 2:** Histopathological evaluation method described by Turkyilmaz et al (2005)

Criteria	Score
Increase in the submucosal collagen	
None	0
Mild^a^	1
Marked^b^	2
Damage to the muscularis mucosa	
None	0
Present	1
Damage and collagen deposition in tunica muscularis	
None	0
Mild^c^	1
Marked^d^	2
Total score	0-5

^a^Submucosal collagen at least twice the thickness of the muscularis mucosa

^b^Submucosal collagen more than twice the thickness of muscularis mucosa

^c^Collagen deposition around the smooth muscle fibers

^d^Same as mild, with collagen deposition replacing some fibers

### Statistical analysis

Comparisons between the groups were performed by using Kruskal-Wallis and post-hoc Dunn tests. *p* Values under 0.05 were accepted as statistically significant. GraphPad Prism version 8 for Windows (GraphPad Software, La Jolla, CA, United States) was used to perform statistical analyses. Statistical powers were calculated by using Power and Sample Size Program (Dupont and Plummer 1998). We calculated the statistical power of comparison between group II and IV regarding the total score as 0.978.

## Results

We used two different approaches to evaluate the extent to which curcumin attenuates the esophageal damage caused by the alkaline substance. Firstly, samples from curcumin-treated groups were compared with the group I samples to assess the level of curcumin’s effect on slowing the progression of esophageal tissue damage. Secondly, the curcumin-treated groups were compared with group II to evaluate the extent of curcumin’s amelioration of esophageal tissue damage.

Samples from group I had normal histological appearance as expected. There was a statistically significant difference between group I and II regarding the level of muscularis mucosa damage (*p*= 0.004), while no difference was found between group I and III as well as group I and IV (*p* values were 0.068 and 0.524, respectively.). Further, there was no statistically significant difference between groups I and IV about the total score (*p*= 0.312), whereas total scores differed significantly among the groups I and II as well as groups I and III. Comparisons between group I and other groups are shown in Table 3.

**Table 3:** *p* Values of post hoc Dunn tests for the comparisons of group I (sham) against other groups

	Group I vs II	Group I vs III	Group I vs IV
Increase in the submucosal collagen	0.001	0.0005	0.009
Damage to the muscularis mucosa	0.004	0.068	0.524
Damage and collagen deposition in tunica muscularis	0.071	0.557	0.999
Total score	0.0004	0.006	0.312

**Fig 1.**
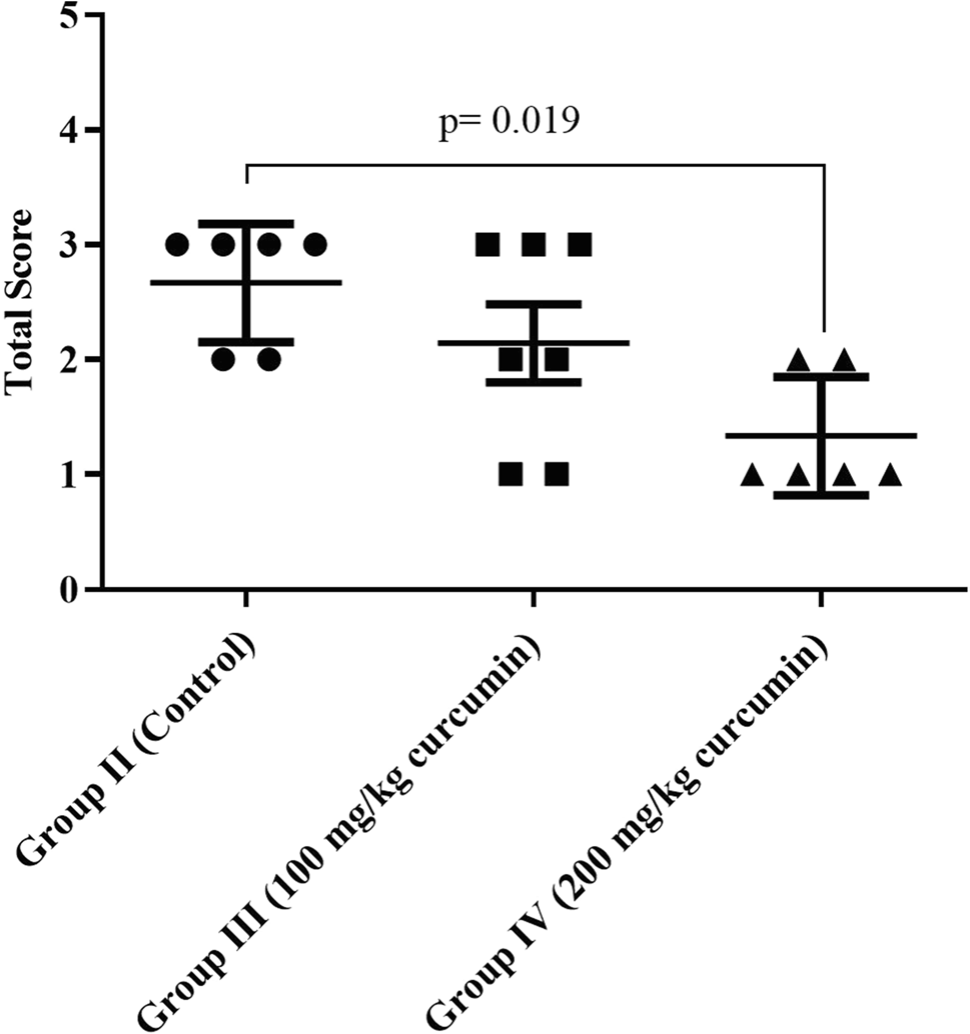
Comparison of total scores between the groups II (control), III (100 mg/kg curcumin) and IV (200 mg/kg curcumin). As mean ± standard deviation (95% confidence interval), total scores were 2.67 ± 0.52 (2.12–3.12), 2.14 ± 0.9 (1.31–2.97), and 1.33 ± 0.52 (1.79–1.88) for the groups II, III, and IV, respectively (*p*= 0.019). There was a statistically significant difference between groups II and IV (adjusted *p*= 0.02).

**Fig 2.**
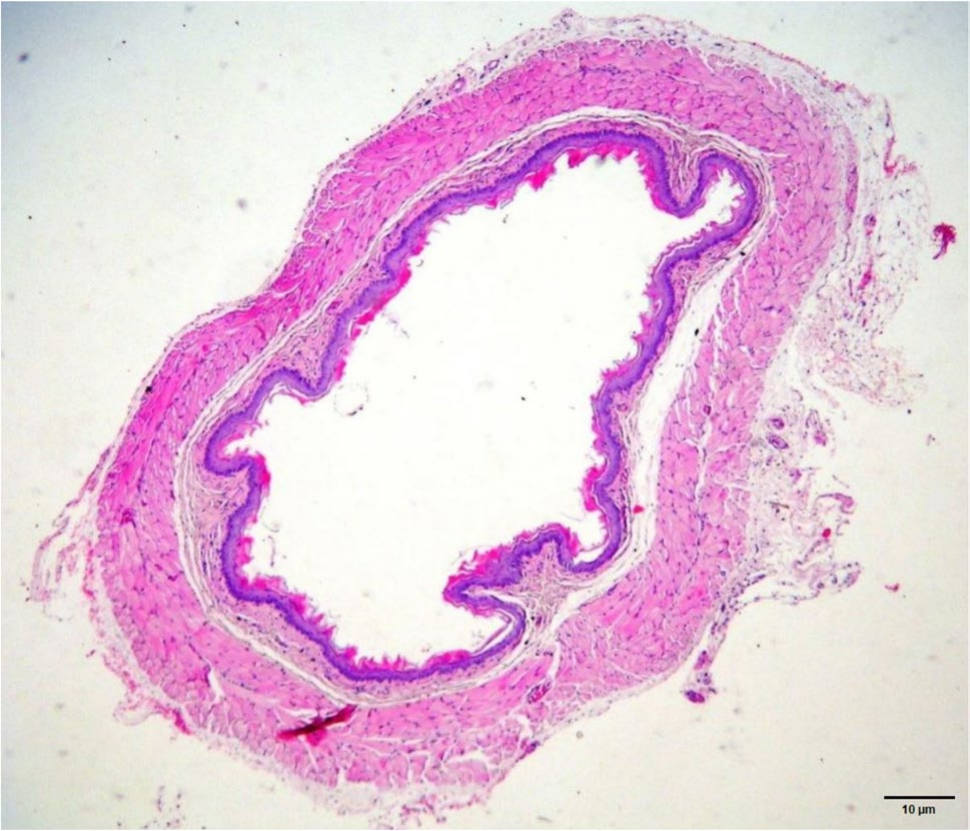
Normal histological appearance from a group I (sham) sample (H&E staining, x40 magnification)

**Fig 3.**
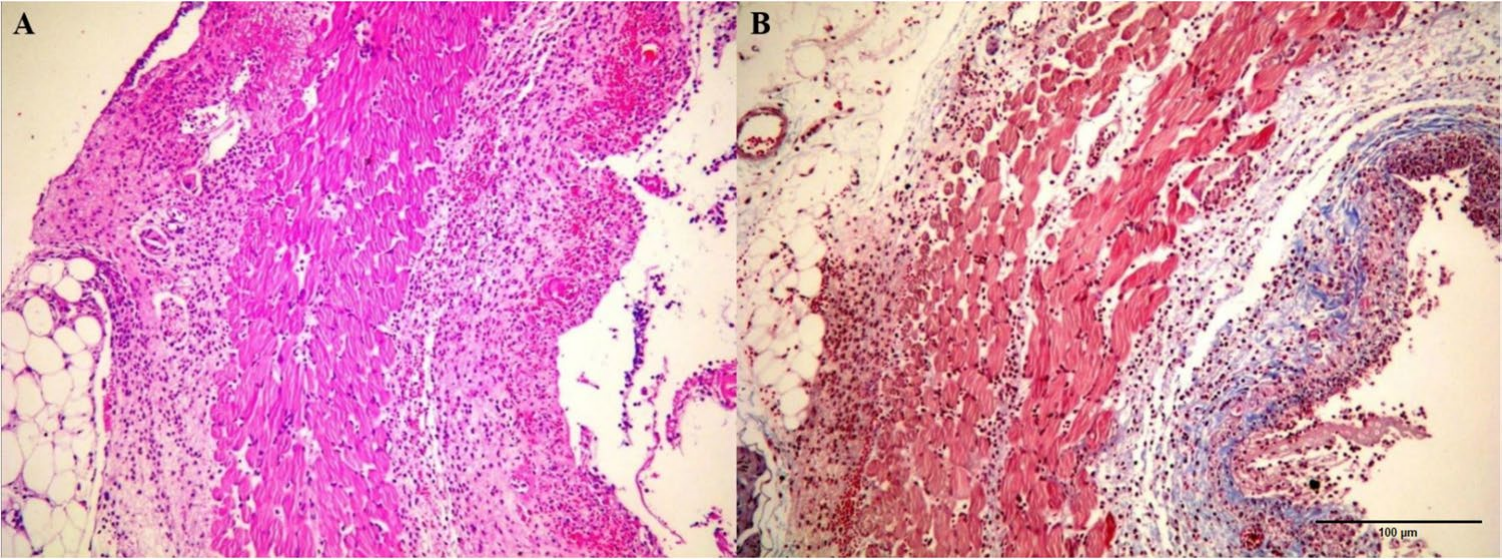
Ulceration and hemorrhage in the surface of esophagus and infiltration of inflammatory cells from a group II (control) sample** (**H&E staining, x100 magnification) (**A**). The ulcerated appearance of the esophagus surface and mild increase in the submucosal collagen from a group II sample (MT staining, x100 magnification) (**B)**

**Fig 4.**
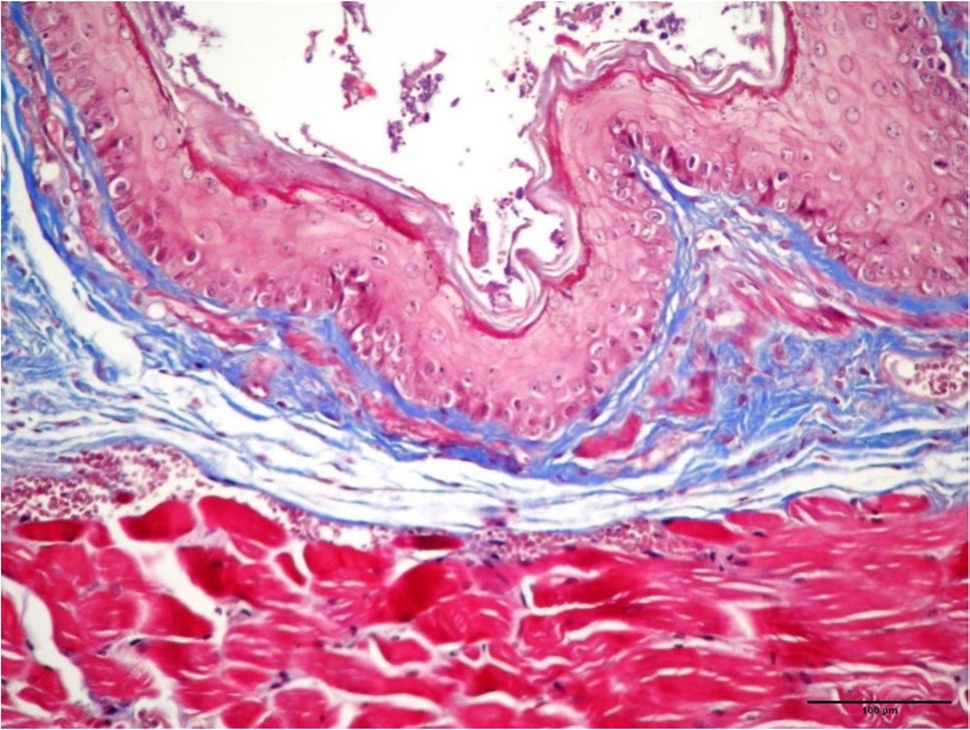
Mild damage and collagen deposition in tunica muscularis from a group III (100 mg/kg curcumin) sample (MT staining, x100 magnification)

**Fig 5.**
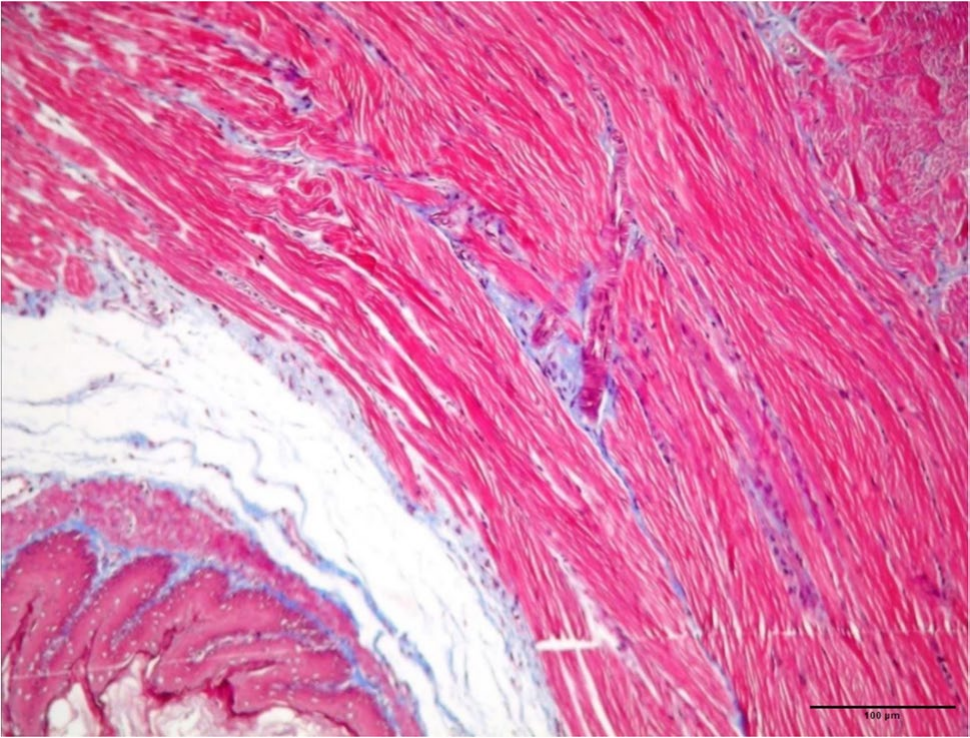
A mild increase in the submucosal collagen from a group IV (200 mg/kg curcumin) sample (MT staining, x100 magnification)

Submucosal collagen increase, muscularis mucosa damage, and collagen deposition in the tunica muscularis were present in all group II samples, while 66.6% of the samples had mild tunica muscularis damage according to both H&E and MT staining. Increase in submucosal collagen was observed in all samples from the first three groups, while in the fourth group, 83.3% of samples had increase in submucosal collagen. Damage to muscularis mucosa was present in all animals in the control group, while this damage was regressed down to 71.4% in group III and 50% in group IV. Further, damage and collagen deposition in the tunica muscularis was regressed from 66.7% of the animals in the control group to 42.9% in group III and to none in group IV. Despite the decrease in the level of tissue damage in a dose-dependent manner; comparisons between the groups II, III, and IV by using Kruskal-Wallis test regarding increase in submucosal collagen, damage to the muscularis mucosa, damage, and collagen deposition in the tunica muscularis yielded no statistically significant results (*p* values were 0.632, 0.196 and 0.066, respectively). However, there was a significant difference between the groups regarding total score (*p*= 0.019). Post hoc analysis revealed that this was resulted from the difference between group II and IV (Fig 1, adjusted *p*= 0.02). Details of the histopathological examination results are shown in Figs. 2, 3, 4, and 5 and Table 4.

**Table 4:** Histopathological examination results from groups II, III, and IV

	Increase in submucosal collagen (0, 1, 2)	Damage to the muscularis mucosa (0, 1)	Damage and collagen deposition in the tunica muscularis (0, 1, 2)	Total score (0–5)
Group II (control)	1 ± 0	1 ± 0	0.67 ± 0.52	2.67 ± 0.57
Group III (100 mg/kg curcumin)	1 ± 0	0.71 ± 0.49	0.43 ± 0.54	2.14 ± 0.9
Group IV (200 mg/kg curcumin)	0.84 ± 0.41	0.5 ± 0.55	0 ± 0	1.33 ± 0.52
*p* Value	0.632	0.196	0.066	0.019

Results are shown as mean ± standard deviation

## Discussion

Our study showed that there were no statistically significant differences between the groups I and III, I, and IV about the level of muscularis mucosa damage, while there was a difference between groups I and II. This finding can be interpreted as alleviation of tissue damage by curcumin in the acute phase of esophagus corrosion due to an alkaline substance. Further, the difference found between groups II and IV regarding total score can be interpreted as such. To our knowledge, this is the first study to report that curcumin reduced the level of esophageal tissue damage due to an alkaline burn in the acute phase.

Despite many clinical studies performed on esophagus corrosion, it still has no conclusive treatment and, it has high frequency of acute and chronic complications. Di Castanzo et al. (1980) reported that, out of 70 patients who applied to an emergency department due to ingestion of an alkaline substance, 7 had deep burns and massive hemorrhage, 6 had ulceration and focal necrosis, 19 had inflammation (Di Costanzo et al. 1980). Further, a previous study that investigated 159 patients by Broto et al. found that 42 patients had first-degree, 22 had second-degree, and 41 had third-degree esophagitis (Broto et al. 1999). Also, various studies in the literature reported a 35% to 85% frequency of esophagitis after corrosive substance ingestion in children (Di Costanzo et al. 1980; Broto et al. 1999; Gun et al. 2007).

Tissue damage caused by alkaline compounds is characterized by inflammation and liquefaction. Inflammation with hemorrhage, thrombosis, and edema are early pathological findings of esophagus corrosion. Within 2 weeks of the injury, thrombosis occurs at submucosal blood vessels, which results in necrosis and edema. In the third week, with the starting of fibroblast accumulation, esophagus stricture begins to develop as proportional to the degree of tissue damage. Stricture is the leading chronic complication of corrosive esophagitis (Desai and Moustarah 2023). A study which was performed by Karnak et al. between the years 1976 and 1995 on 282 patients showed that 67% of the patients who suffered from esophagus corrosion due to alkaline substance ingestion developed esophagus stricture (Karnak et al. 1999). Sixty percent to 85% of the esophagus stricture frequency after corrosive substance ingestion were reported by other studies in the literature (Han et al. 2004). The mentioned studies also reported that stricture development was the most important factor affecting morbidity and mortality after esophagus corrosion. Most importantly, previous studies concluded that early phase tissue damage level, which is associated with severity of inflammation, was associated with later phase stricture development (Gunnarsson 1999).

There has been no established treatment protocol for the treatment of esophagus corrosion and, various supportive treatment approaches which differ according to the severity of the corrosion are used (O’Malley and O’Malley 2023). Antibiotics and steroids are widely used against stricture development. Drug doses and duration of treatments differ between physicians (Karnak et al. 1999).

Different treatment modalities were investigated in the literature. β-Aminopropionitrile and penicillamine were found to be more effective than steroids against esophageal stricture development after caustic ingestion in animal studies (Aciksari et al. 2013; Gehanno and Guedon 1981). However, no further clinical trials were conducted investigating these two agents. Similarly, estradiol and progesterone were found to reduce stricture development by inhibiting collagen synthesis. Nevertheless, physicians do not use these two hormones to alleviate stricture development after corrosive substance ingestion because of their side effects of inhibition of endogen estradiol and progesterone synthesis (Liman et al. 2005). Anticoagulant agent heparin was also investigated and found to be effective against severe strictures, but its use is limited by its side effect of hemorrhage (Bingol-Kologlu et al. 1999).

Curcumin shows anti-inflammatory effects by downregulating NLRP3 inflammasomes; suppressing NF‐κB signaling pathway; decreasing COX-2 activity; and reducing the levels of proinflammatory cytokines such as IL‐1β, IL‐6, IL‐18, and TNF‐α (8). Anti-inflammatory effects of curcumin were found to be beneficial in animal models of inflammatory diseases such as gout (Li et al. 2019). Further, the same beneficial anti-inflammatory effects have been found to show a mucosal protective effect against colitis, which is an inflammatory disease of the gastrointestinal mucosa (Gong et al. 2018). Also, the cell-protective effect of curcumin by modulating pro-inflammatory cytokines has been demonstrated in conditions that do not progress mainly with inflammation such as ischemic brain injury (Li et al. 2015). Our finding of curcumin’s alleviating effect against corrosive tissue damage in esophagus at a dose of 200 mg/kg was in line with mentioned studies that investigated the effects of curcumin in other tissues. We are aware that the anti-inflammatory and cell-protective effects of curcumin which is shown in other tissues including a type of gastrointestinal tissue, colon, cannot be directly interpreted as such in the esophageal tissue and rat esophageal tissue differs from human esophageal tissue. However, suppression of the pro-inflammatory pathways and proteins by curcumin has been shown to protect cells in similar conditions in animals as well as in humans (Hasanzadeh et al. 2020). Curcumin may be beneficial against corrosive esophagitis in the early phase through its anti-inflammatory activity. Furthermore, curcumin may alleviate the severity of later phase stricture development, which is associated with the level of early phase esophagus tissue damage (Gunnarsson 1999) and is the main complication of corrosive esophagitis, because it may suppress inflammation in the early phase. In addition, therapeutic doses of curcumin have no serious side effects in humans (Hasanzadeh et al. 2020), unlike some other agents which were investigated for the treatment of corrosive esophagitis such as penicillamine (Gehanno and Guedon 1981). Despite its low bioavailability in certain organs, curcumin can reach gastrointestinal organs in high concentrations (Garcea et al. 2005). This can be considered an advantage in the case of esophagitis. Therefore, curcumin may have the potential to be a supportive treatment for corrosive esophagitis in the acute phase if the result of the present study has to be confirmed by future studies (Hasanzadeh et al. 2020). We suggest furthering the clinical research on curcumin’s use in esophageal burns according to our results.

There were limitations in our study. We could not manage to follow-up animals more than 24 h, and we could manage to evaluate pro-inflammatory cytokines among the groups. We accept that our results are not conclusive. Nevertheless, we think that our results have the potential to contribute to the existing literature.

## Conclusions

Curcumin decreased the level of esophageal tissue damage due to an alkaline substance ingestion in the acute phase at a dose of 200 mg/kg/day. With the confirmation of our results by future clinical studies, curcumin may be considered a supportive treatment for acute corrosive esophagitis.

## Data Availability

All data generated or analyzed during this study are included in this article.
